# *In vivo* Evaluation of Two Thiazolidin-4-one Derivatives in High Sucrose Diet Fed Pre-diabetic Mice and Their Modulatory Effect on AMPK, Akt and p38 MAP Kinase in L6 Cells

**DOI:** 10.3389/fphar.2016.00381

**Published:** 2016-10-14

**Authors:** Jayesh Mudgal, Priya Shetty, Neetinkumar D. Reddy, H. S. Akhila, Karthik Gourishetti, Geetha Mathew, Pawan G. Nayak, Nitesh Kumar, Anoop Kishore, Nampurath G. Kutty, Krishnadas Nandakumar, Rekha R. Shenoy, Chamallamudi M. Rao, Alex Joseph

**Affiliations:** ^1^Department of Pharmacology, Manipal College of Pharmaceutical Sciences, Manipal UniversityManipal, India; ^2^Department of Pharmacy Practice, Manipal College of Pharmaceutical Sciences, Manipal UniversityManipal, India; ^3^Department of Pharmaceutical Chemistry, Manipal College of Pharmaceutical Sciences, Manipal UniversityManipal, India

**Keywords:** thiazolidin-4-one derivatives, high sucrose diet, mouse model, AMPK, Akt, p38 MAP kinase

## Abstract

We had previously demonstrated the anti-diabetic potential and pancreatic protection of two thiazolidin-4-one derivatives containing nicotinamide moiety (NAT-1 and NAT-2) in STZ-induced diabetic mice. However, due to the limitations of the STZ model, we decided to undertake a detailed evaluation of anti-diabetic potential of the molecules on a high sucrose diet (HSD) fed diabetic mouse model. Further, *in vitro* mechanistic studies on the phosphorylation of AMPK, Akt and p38 MAP kinase in L6 myotubes and anti-inflammatory studies in RAW264.7 mouse monocyte macrophage cells were performed. 15 months of HSD induced fasting hyperglycaemia and impaired glucose tolerance in mice. Treatment with NAT-1 and NAT-2 (100 mg/kg) for 45 days significantly improved the glucose tolerance and lowered fasting blood glucose levels compared to untreated control. An improvement in the elevated triglycerides and total cholesterol levels, and favorable rise in HDL cholesterol were also observed with test drug treatment. Also, no major changes were observed in the liver (albumin, AST and ALT) and kidney (creatinine and urea) parameters. This was further confirmed in their respective histology profiles which revealed no gross morphological changes. In L6 cells, significant phosphorylation of Akt and p38 MAP kinase proteins were observed with 100 μM of NAT-1 and NAT-2 with no significant changes in phosphorylation of AMPK. The molecules failed to exhibit anti-inflammatory activity as observed by their effect on the generation of ROS and nitrite, and nuclear levels of NF-κB in LPS-stimulated RAW264.7 cells. In summary, the molecules activated Akt and p38 MAP kinase which could have partly contributed to their anti-hyperglycaemic and hypolipidemic activities *in vivo*.

## Introduction

We had previously reported pancreatic islet cell protection in streptozotocin (STZ)-induced diabetic mice by two nicotinic acid derivatives of thiazolidin-4-ones (designated NAT-1 and NAT-2; Kishore et al., 2009). The molecules had exhibited anti-hyperglycaemic and hypolipidemic activities in animal models of diabetes and hyperlipidaemia, and reversed the damage to the islets induced by streptozotocin (Joy et al., 2005; Nampurath et al., 2008; Kishore et al., 2009). Though the STZ model of diabetes in rodents, induced by destruction of islets of pancreas (Szkudelski, 2001; Chatzigeorgiou et al., 2009), is a widely reported, fast, easy, and convenient method to evaluate anti-hyperglycaemic activities of test compounds, the etiology of human type-II diabetes mellitus (T2DM) seldom resembles this acute preclinical model. Most cases of human T2DM involves chronic insulin resistance, hyperinsulinemia, dyslipidemia, and hyperglycaemia that eventually lead to pancreatic dysfunction (Defronzo and Ferrannini, 1991). These conditions usually arise out of an interplay between genetic and environmental factors with lifestyle and food habits assuming important roles. So it was decided to test the molecules on a model of diabetes induced by chronic feeding of diet containing higher proportions of sugars and fats (Sumiyoshi et al., 2006). In the current study, the effect of the molecules were evaluated in mice rendered pre-diabetic by feeding a diet containing high sucrose (55%) for a duration of 15 months.

In our previous studies, it was observed that the anti-hyperglycaemic activities of the molecules were in part due to their ability to enhance glucose uptake possibly through the modulation of glucose metabolic pathway. Akt, AMPK, and p38 MAP kinase are key components of the metabolic pathways that modulate glucose uptake independent of one another. The binding of insulin to its receptor leads to the phosphorylation of Akt. This eventually facilitates the translocation of GLUT4 transporters to the cell membrane leading to the uptake of glucose in cells (Kohn et al., 1996; Manning and Cantley, 2007). Phosphorylated Akt also modulates glycogen synthase kinase (GSK-3) that exerts control over glucose metabolism (Cross et al., 1995). AMPK, a cellular fuel gauge activated in response to metabolic stress like energy starvation resulting in higher AMP/ATP ratios, plays important roles in lipid and glucose homeostasis. Phosphorylation of AMPK, through non-insulin dependent pathways, also triggers GLUT4 translocation and glucose uptake in skeletal muscle and adipocytes, increased fatty acid oxidation and decreased fatty acid synthesis in liver and skeletal muscles improving insulin sensitivity. It also decreases cholesterol synthesis and gluconeogenesis in liver (Carling, 2004; Towler and Hardie, 2007; Zhang et al., 2009). The p38 mitogen activated protein kinase (p38 MAP kinase), a stress-activated serine/threonine protein kinase which is activated by a wide variety of chemicals including insulin, stimulates glucose uptake independent of AMPK and Akt pathway. It also controls inflammation and biosynthesis of proinflammatory cytokines like IL-1 and TNF-α that play an important role in the development of insulin resistance (Chambers et al., 2009; Kim and Choi, 2010). Therefore, *in vitro* investigations were carried out to evaluate the effect of the molecules on a few key components of the metabolic pathways like AMPK, Akt, and p38 MAP kinase in L6 myotubes.

Inflammation plays an important role in the development of diabetes. Chronic inflammation originating in adipose tissues under conditions of hyperlipidaemia causes increased infiltration of macrophages. Chronic activation of these macrophages and their subsequent release of adipo-cytokines like TNF-α, IL-6, IL-1 and activation of NF-κB eventually cause insulin resistance in skeletal muscles (Xu et al., 2003; Kristiansen and Mandrup-Poulsen, 2005; Nieto-Vazquez et al., 2008). Since the mechanism of islet destruction by STZ is partly due to inflammation, and as our molecules had previously shown partial reversal of islet damage, it was also decided to evaluate the anti-inflammatory activities of the molecules, by evaluating their effect on nitric oxide (NO) and reactive oxygen species (ROS) production and NF-κB levels in lipopolysaccharide (LPS) treated RAW264.7 (mouse monocyte macrophages) cells.

In the current study, both test molecules, NAT-1 and NAT-2, were able to improve the glucose tolerance in 15 months HSD-fed mice. In addition, no major liver or kidney toxicities were observed due to treatments. NAT-2 showed increased phosphorylation of Akt in L6 cells suggesting a possible role in Akt-mediated glucose uptake.

## Materials and Methods

### Chemicals

**FIGURE 1:**
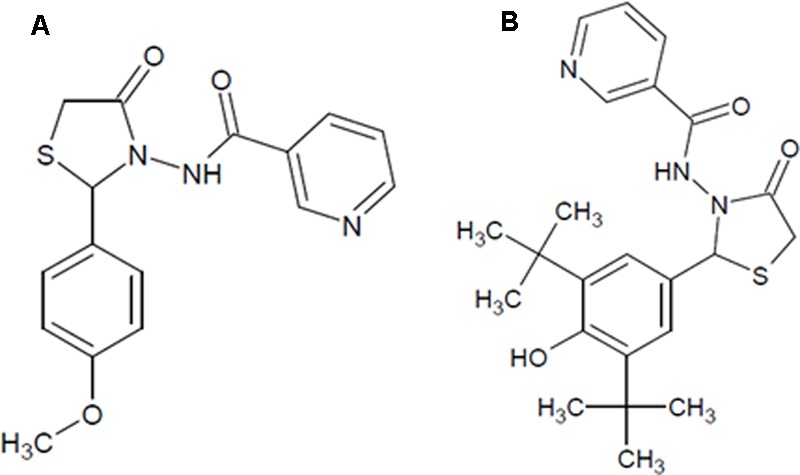
**The chemical structure of the test compounds. (A)** NAT-1: N-[2-(4-Methoxy-phenyl)-4-oxo-thiazolidin-3-yl]-nicotinamide; **(B)** NAT-2: N-[2-(3,5-Di-*tert*-butyl-4-hydroxy-phenyl)-4-oxo-thiazolidin-3-yl]-nicotinamide.

The molecules NAT-1 and NAT-2 (**Figure 1**) were synthesized, purified and characterized as explained previously (Kishore et al., 2009). Fetal bovine serum (Gibco^TM^) was purchased from Thermo Fisher Scientific Inc. (Waltham, MA, USA). Casein, cellulose, methionine, Dulbecco’s Modified Eagle Medium (DMEM), penicillin–streptomycin, and trypsin–EDTA were purchased from HiMedia Laboratories (Mumbai, Maharashtra, India). Primary antibodies of AMPKα, Phospho-AMPKα (Thr172), Akt, Phospho-Akt (Ser473), p38 MAP kinase (MAPK), Phospho-p38 MAPK (Thr180/Tyr182), Phospho NF-kB were purchased from Cell Signaling Technology (Danvers, MA, USA). Cholesterol, choline bitartrate were procured from Sigma-Aldrich Co. LLC (St. Louis, MO, USA). Normal pellet diet for the animals were purchased from Amrit Feeds Ltd, Pune, Maharashtra, India. Sugar (Madhur brand) manufactured by Shree Renuka Sugars Ltd (Belgaum, Karnataka, India) was procured from the local market. The kits for biochemical estimations like triglycerides, total cholesterol, HDL cholesterol were purchased from Rapid Diagnostic Pvt. Ltd (Aspen Kits, New Delhi, India), and kits for the estimation of AST, ALT, albumin, creatinine and urea were purchased from Roche Diagnostics Ltd (Basel, Switzerland).

### Animals and Treatment

The studies on whole animals and animal tissue samples were conducted after obtaining clearance from the Institutional Animal Ethics Committee, Kasturba Medical College, Manipal University (approval no. IAEC/KMC/72/2012).

Male Swiss albino mice aged 3–4 weeks old and weighing 15–20 g were used for the study. The animals were maintained in polypropylene cages in the Central Animal Research Facility of Manipal University, with controlled temperature and 12 h light–dark cycle. Four animals were housed per cage, and maintained on high sucrose diet (HSD) (containing normal pellet diet 28.1%, Sucrose 55%, Casein 8%, Cellulose 5%, Fats 3%, Methionine 0.5%, Choline bitartrate 0.25%, Mineral mix 0.15%) and water *ad libitum* for 15 months. Another group of mice (*n* = 6) were fed normal pellet diet that served as normal control.

At the end of 15 months, the HSD-fed mice were anesthetized using isoflurane (Forane^®^, Abbott House, Berkshire, UK) and 0.5 ml of blood was withdrawn retro-orbitally using micro haematocrit tubes (Brand GMBH + CO KG, Wertheim, Germany) in a 1.5 ml micro-centrifuge tube (Tarsons Products Pvt. Ltd, Kolkata, West Bengal, India) containing dipotassium EDTA (2 mg/ml of blood). Plasma was separated by centrifuging (Micro 22R, Andreas Hettich GmbH & Co. KG, Tuttlingen, Germany) at 6,000 rpm for 5 min at 25°C. The plasma glucose levels were estimated and only those animals with fasting plasma glucose of 120–200 mg/dl (pre-diabetic) were included in the study.

The pre-diabetic animals were randomized into four groups, three treatment groups and one control group (*n* = 5–6), with the same mean blood glucose.

Test compounds, NAT 1 and NAT-2, were prepared as suspensions in 0.3% carboxy-methyl cellulose (CMC) and administered in a dose of 100 mg/kg of body weight. These doses were selected based on previous studies conducted on these compounds (Kishore et al., 2009). The standard drug metformin was also prepared similarly as a suspension in CMC and administered in a dose of 200 mg/kg body weight. The pre-diabetic control group and normal control group received only 0.3% CMC suspension. All the treatments were given once daily orally for 45 days, during which period the animals were maintained on their respective diets. On day 46, an oral glucose tolerance test (OGTT) was performed on all the mice and the animals were anesthetized and blood withdrawn by retro-orbital puncture. Plasma levels of triglycerides, total cholesterol, HDL cholesterol were estimated colourimetrically using Star 21 semi-automatic analyser (Rapid Diagnostic Pvt. Ltd, New Delhi, India) and the levels of AST, ALT, albumin, creatinine and urea was estimated on Cobas^®^ C 111 analyzer (Roche Diagnostics, Basel, Switzerland). The animals were then sacrificed under excess anesthesia, and the liver and kidneys collected in 10% formalin, sectioned and stained using H&E stain (hematoxylin and eosin). The histopathological assessment was done by an investigator blinded to the treatment.

### Oral Glucose Tolerance Test (OGTT)

Oral glucose tolerance test was performed on mice fasted for 6 h (Andrikopoulos et al., 2008). Glucose levels of blood taken from the tip of tail were estimated using a glucometer (Accu-Chek^®^ Sensor Comfort, Roche Diagnostics India Pvt. Ltd, Mumbai, Maharashtra, India) to obtain the basal glucose levels. Then, glucose (2 g/kg, b.w.) was administered orally and blood glucose levels were again measured at 5, 15, 30, 60, and 120 min of glucose administration. Drugs were administered orally 1 h before glucose administration.

### Cell Culture and Maintenance

L6 myoblasts (rat muscles) and RAW264.7 (mouse monocyte macrophage) cells were procured from National Centre for Cell Sciences, Pune, Maharashtra, India and maintained in cell culture flasks (25 or 75 cm^2^) containing Dulbecco’s modified Eagle’s medium (DMEM) complete culture medium (90% DMEM and 10% fetal bovine serum) with 1% penicillin-streptomycin at 37°C in a 5% carbon dioxide atmosphere (CO_2_ incubator, NU-5510E, NuAire Inc., Plymouth, MN, USA). After becoming confluent, the cells were trypsinized and sub-cultured into fresh flasks. For all studies, cells between passages 15 and 20 were used. L6 myotubes were obtained by differentiation of myoblasts by modification of method explained elsewhere (Mitsumoto and Klip, 1992). All the treatments were done using test compounds solubilized in 0.1% DMSO.

### MTT Assay

Briefly, 100 μl of cell suspension containing 5 × 10^3^ cells (L6 or RAW264.7) was added in each well of a sterile 96-well flat bottom tissue culture plate. Twenty four hours later, cells were treated with different concentrations (25–200 μM) of test compounds and incubated for 48 h. The control group was treated only with medium containing 0.1% DMSO. Then, 30 μl of MTT (3-(4,5-dimethyl-2-thiazoyl)-2,5-diphenyl-2H-tetrazolium bromide) reagent (4 mg/ml) was added in each well and incubated at room temperature for 4 h. Later, the medium was removed and 100 μl of DMSO was added to each well and absorbance was measured using a microplate reader (ELx800, Biotek Instrument Inc., Winooski, VT, USA) at 540 nm and IC_50_ values of the compounds were determined (Mosmann, 1983).

### Estimation of ROS and NO, and Nuclear Levels of NF-κB in LPS Stimulated RAW 264.7 Macrophages

Previously reported method was used for ROS and NO estimation (Mathew et al., 2013). Briefly, cell suspension (100 μl), containing 5 × 10^4^ RAW264.7 cells was seeded in a 96-well black plate. Twenty four hours later, different concentrations of standard, L-NAME hydrochloride (*N*^G^-nitro-_L_-arginine methyl ester) and DPI (diphenylene iodonium) for NO and ROS, respectively, and test drugs were added to the wells. One hour after drug addition, 100 μl of LPS (10 μg/ml) was added into each of the wells and incubated for 20 h. The supernatant was aspirated from each well and used for nitrite assay. Then to each of the wells, 100 μM of DCFH-DA (dichloro-dihydro-fluorescein diacetate) was added and incubated for 1 h, washed with freshly prepared Hank’s balanced salt solution and fluorescence intensity measured using a fluorescence microplate reader (FLx800, Biotek Instrument Inc., Winooski, VT, USA) at λ_ex_: 485 nm and λ_em_: 530 nm. For nitrite estimation, 100 μl of freshly prepared Griess reagent was added to the aspirated supernatant, incubated at room temperature for 10 min and absorbance measured at 540 nm. The experiment was performed in triplicate.

For measuring the levels of NF-κB, 4 × 10^6^ RAW264.7 cells seeded in 75 cm^2^ cell culture flasks were treated with 100 μM of test drugs and stimulated with LPS as mentioned above. After the cells were washed, the nuclear fraction was prepared using commercially available nuclear extraction kit (Cayman Chemical Company, Ann Arbor, MI, USA) and stored at -80°C until estimation of NF-κB by Western blot.

### Effect of Test Compounds on Phosphorylation of AMPK, Akt and p38 MAP Kinase in L6 Myotubes

L6 myoblast cells (2 × 10^6^), seeded in 75 cm^2^ cell culture flasks, were differentiated into myotubes, as described elsewhere (Mitsumoto and Klip, 1992; Yap et al., 2007). The media in the flasks were replaced with glucose-free DMEM and incubated with two concentrations (10 and 100 μM) of compounds (metformin, NAT-1 and NAT-2). Four hours later, the cells were washed with PBS, scraped, lysed, protein content estimated and subsequently used for Western blot.

### Western Blot Analysis

The protein content of the cell lysates/ fractions was determined using a bicinchoninic acid assay (BCA^TM^ protein assay kit, Thermo Fisher Scientific Inc., Waltham, MA, USA). The lysates were adjusted to 20–25 μg of protein and was resolved in 10% SDS-polyacrylamide gel and transferred to a polyvinylidene difluoride (PVDF) membrane. Then the membrane was blocked for 1 h at room temperature with 5% non-fat milk protein in Tris buffered saline (TBS) containing 0.1% Tween 20. The blot was incubated with 1:1000 dilutions of primary antibodies of interest and the GAPDH antibody (housekeeping standard) over-night at 4°C. The blot was then washed and incubated with a 1:1000 dilution of IgG–horseradish peroxidase (HRP) conjugate. For estimation of NF-κB, amplified Opti-4CN substrate kit (Bio-Rad Laboratories Inc., Hercules, CA, USA) was used to visualize the proteins and image captured using a calibrated densitometer (GS 800, Bio-Rad Laboratories Inc., Hercules, CA, USA). For estimation of metabolic markers, signal was captured using SignalFire^TM^ Plus ECL reagent kit (#12630S, Cell Signaling Technology Inc., Danvers, MA, USA) on a gel documentation system (G:BOX Chemi XRQ, Syngene, A Division of Synoptics Ltd, Cambridge, UK). The experiments were performed in triplicate. Protein band densities were estimated using the software ImageJ (version 1.46r, NIH, Bethesda, MD, USA).

### Statistical Analysis

The values are given as mean + SEM. The oral glucose tolerance is expressed as area under the curve (AUC) in arbitrary units. The levels of NF-κB is expressed as the relative band density (i.e., ratios of band intensities of NF-κB to that of its respective GAPDH band). The phosphorylation of AMPK, Akt, and p38 MAP kinase are expressed as the ratio of relative densities of phosphorylated and non-phosphorylated proteins with respect to their corresponding housekeeping protein. The comparison of glucose and TG levels between normal and HSD diet animals after 15 months of feeding was analyzed by unpaired *t*-test. All other results were analyzed by one way ANOVA followed by Tukey’s *post hoc* analysis using Prism fully functional demo version (version 6.01, GraphPad Software Inc., La Jolla, CA, USA). *p* < 0.05 was considered statistically significant.

## Results

### Effect of 15-month High-Sucrose Diet on Fasting Blood Glucose and Triglyceride Levels in Mice

**FIGURE 2:**
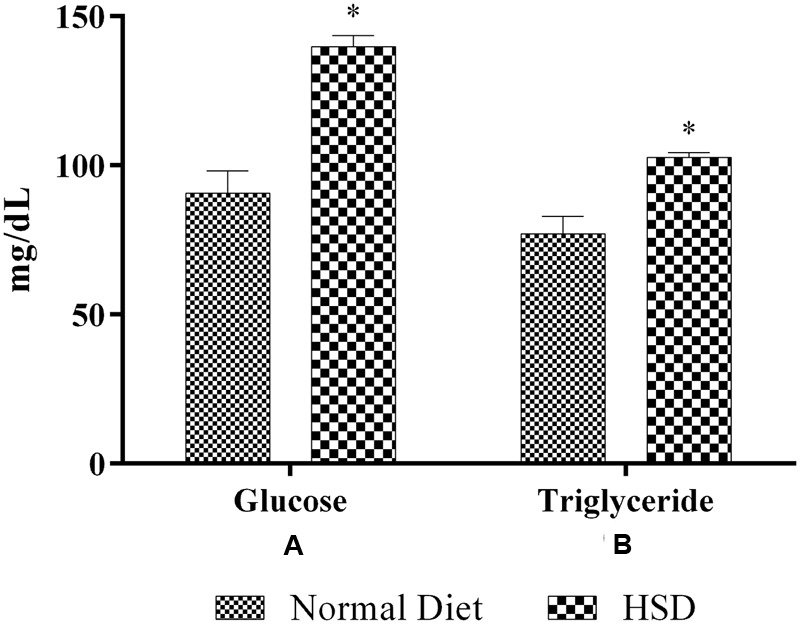
**Effect of 15-month high-sucrose diet on** **(A)** fasting blood glucose; **(B)** plasma triglyceride levels in mice. All values are expressed as mean + SEM (*n* = 6 for normal animals and 21 for HSD fed animals). ^∗^*p* < 0.05 compared to normal diet.

After 15-month HSD, there was a significant increase (*p* < 0.05) in the mean fasting blood glucose and triglyceride levels in the mice fed the diet, compared to normal animals which were fed standard rodent diet (**Figure 2**).

### Effect of 45 Days of NAT Treatment on OGTT in HSD Fed Mice

**FIGURE 3:**
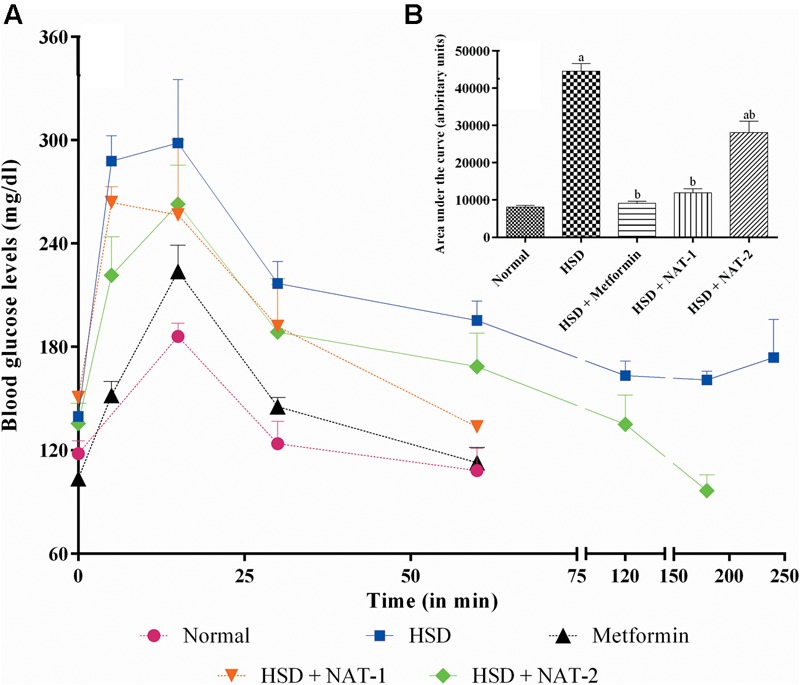
**Effect of 15-month high sucrose diet (HSD) on OGTT after a glucose load (2 g/kg, b.w.), where** **(A)** plot of plasma glucose levels (mg/dl) vs. time interval; **(B)** area under the curve (AUC; expressed as arbitrary units) of the glucose tolerance curve. The time taken for the plasma glucose levels to come back to their pre-glucose-load values were considered for calculation of AUC. All values are expressed as mean + SEM (*n* = 5–6). ^a^*p* < 0.05 compared to normal control; ^b^*p* < 0.05 compared to HSD control. In OGTT, after the glucose load in the normal diet fed group, the basal plasma glucose levels were attained in less than 60 min whereas in HSD fed group, the basal levels were was not attained even after 4 h. With metformin and NAT-1 treatment, the basal blood glucose was attained in 60 min and in 2 h by NAT-2 treatment.

High sucrose diet significantly altered the glucose tolerance profile in mice (**Figure 3**). The mean AUC of the HSD control group was significantly higher than normal diet fed group. Treatment for 45 days with metformin, NAT-1 and NAT-2 significantly (*p* < 0.05) reduced the AUC in HSD fed mice compared to HSD control (**Figure 3B**).

After the glucose load, in animals fed normal pellet diet, blood glucose levels came back to the pre-glucose load (basal) values in 1h whereas in HSD fed group, the basal level was not attained even after 4 h. In the metformin and NAT-1 treated animals, the basal glucose levels were attained in hr and in the NAT-2 treated group glucose levels came back to the initial values by 2 h. The effect of NAT-1 was comparable with that of the standard drug (**Figure 3A**).

### Effect of 45 Days of NAT-1 and NAT-2 Treatments on Lipid Parameters in HSD Fed Mice

**FIGURE 4:**
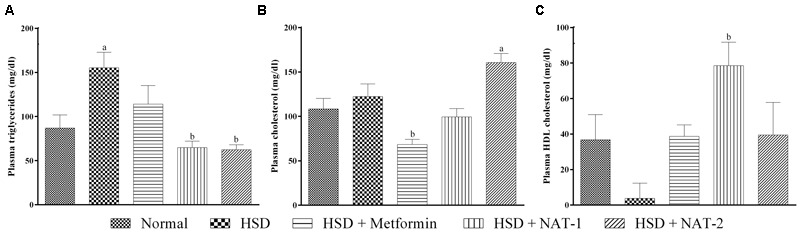
**Effect of NAT-1 and NAT-2 on lipid parameters in plasma of HSD-fed mice. (A)** Triglyceride levels; **(B)** total cholesterol levels; **(C)** HDL cholesterol levels. All values are expressed as mean ± SEM (*n* = 5–6). ^a^*p* < 0.05 vs. normal control, ^b^*p* < 0.05 vs. HSD control.

There was a significant rise in the triglyceride levels in HSD fed mice when compared to the normal diet fed animals (**Figure 4A**). NAT-1 and NAT-2 treatments produced a significant reduction in the elevated TG levels in HSD fed animals.

The cholesterol levels did not rise significantly in HSD fed mice compared to the normal diet fed animals (**Figure 4B**). A significant rise in cholesterol levels was observed with NAT-2 treatment compared to normal animals.

Treatment with NAT-1 led to a statistically significant increase in the HDL cholesterol level compared to HSD fed animals (**Figure 4C**).

### Effect of 45 Days of NAT-1 and NAT-2 Treatments on Plasma Albumin, AST and ALT Levels, and Liver Tissue in HSD Fed Mice

**FIGURE 5:**
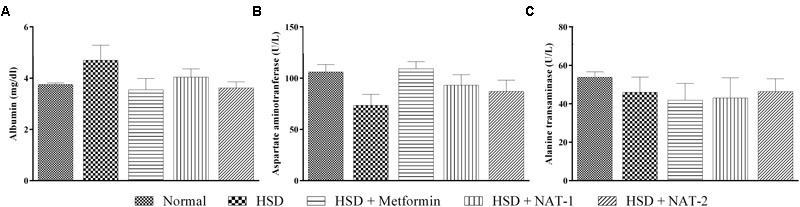
**Effect of NAT-1 and NAT-2 on liver parameters in plasma of HSD-fed mice. (A)** Albumin levels; **(B)** aspartate aminotransferase (AST) levels; **(C)** alanine transaminase (ALT) levels. All values are expressed as mean ± SEM (*n* = 5–6). No significant difference was observed.

**FIGURE 6:**
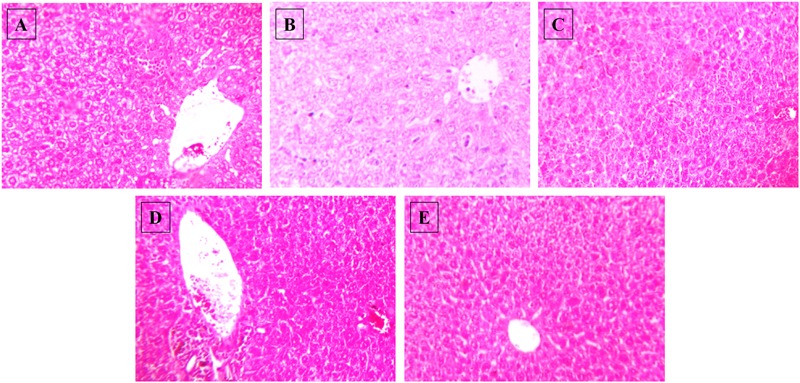
**Effect of NAT-1 and NAT-2 on morphological feature of mice liver.** Optical microscopy: H&E (200×). Photomicrographs of representative mice are shown. **(A)** Normal control; **(B)** HSD fed control; **(C)** HSD fed mice treated with metformin; **(D)** HSD fed mice treated with NAT-1; **(E)** HSD fed mice treated with NAT-2.

There were no significant differences observed in the mean plasma levels of albumin, AST and ALT between the normal control group and HSD control (**Figure 5**). Similarly, no changes were observed in any of the treatment groups. In the HSD group, liver histology showed focal fatty lobules, lymphocytic infiltration in lobules and focal dilation of sinusoidal spaces whereas in the treatment groups only fatty lobules were observed. There was no fibrosis or steatosis or necrosis observed in any of the groups (**Figure 6**).

### Effect of 45 Days of NAT-1 and NAT-2 Treatments on Plasma Creatinine and Urea Levels, and Kidney Tissue in HSD Fed Mice

**FIGURE 7:**
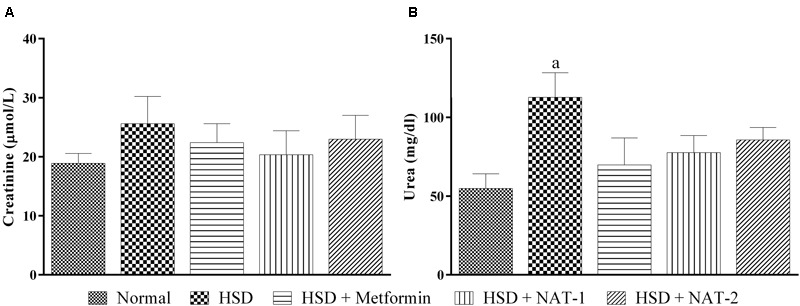
**Effect of NAT-1 and NAT-2 on kidney parameters in plasma of HSD-fed mice. (A)** Creatinine levels; **(B)** urea levels. All values are expressed as mean ± SEM (*n* = 5–6). ^a^*p* < 0.05 vs. normal control.

**FIGURE 8:**
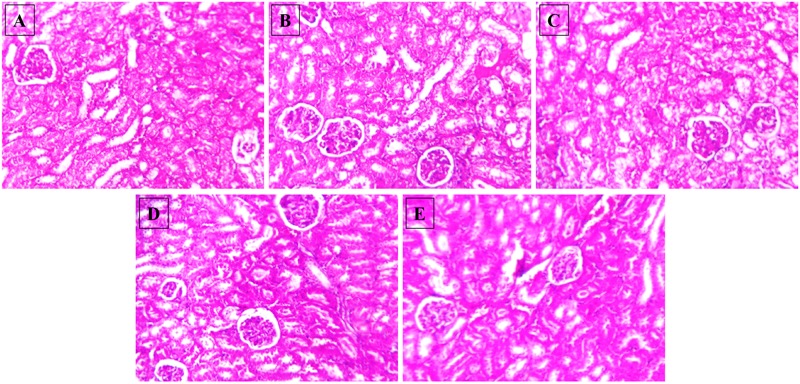
**Effect of NAT-1 and NAT-2 on morphological feature of mice kidney.** Optical microscopy: H&E (200×). Photomicrographs of representative mice are shown. **(A)** Normal control; **(B)** HSD fed control; **(C)** HSD fed mice treated with metformin; **(D)** HSD fed mice treated with NAT-1; **(E)** HSD fed mice treated with NAT-2.

After 15-month HSD, there was a significant increase in the mean plasma urea level in the mice fed the diet, compared to the animals on normal pellet diet. There were no significant differences in the mean urea levels of any of the treatment groups and the animals fed normal pellet diet (**Figure 7B**). No significant differences in the plasma mean creatinine levels were observed in any of the treatment groups (**Figure 7A**). Histology of kidney in the HSD fed mice showed focal crowding of glomeruli and lymphocytic infiltration. Whereas, in all treatment groups only lymphocytic infiltration was observed (**Figure 8**).

### Intrinsic Toxicity of the Test Compounds in Cell Lines

To assess the effect of the test molecules on the activation of the components of metabolic signaling, we first evaluated the intrinsic toxicity of the molecules by MTT assay in L6 and RAW264.7 cells. NAT-1 and NAT-2 were found to elicit low level cytotoxicity as observed by the IC_50_ values (μM) of 1654.62 ± 158.77 and 684.18 ± 114.27 in L6 cells and 1436.62 ± 132.62 and 751.25 ± 74.23 in RAW264.7 cells, respectively (data not shown). Based on the results, it was decided to carry out all *in vitro* studies using two doses (100 and 200 μM) of the test compounds.

### Effect of NAT-1 and NAT-2 on NO and ROS Generation, and Levels of NF-κB in LPS Stimulated RAW264.7 Cells

**FIGURE 9:**
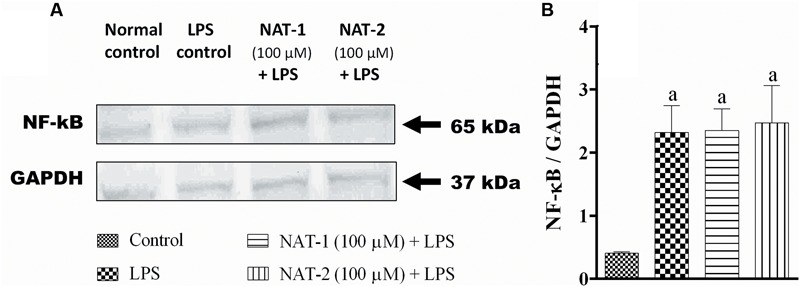
**Effect of NAT-1 and NAT-2 on nuclear levels of NF-κB in LPS stimulated RAW264.7 cells where** **(A)** blot showing nuclear levels of NF-κB; **(B)** relative density ratio of NF-κB/GAPDH. Values are expressed as mean ± SEM. ^a^*p* < 0.05 vs. normal control.

The compounds were found to have very little effect on the inhibition of ROS and NO production, and reduction of NF-κB level in LPS-stimulated RAW264.7 cells. L-NAME hydrochloride, a NO synthase inhibitor, gave an IC_50_ value of 156.28 ± 2.03 μM whereas in both the test molecules the IC_50_ values were more than 600 μM (highest concentration tested) in NO inhibition assay (data not shown). Similarly, DPI, an NAD(P)H oxidase inhibitor, inhibited ROS production with an IC_50_ of 4.29 ± 3.53 μM but both the test molecules were unable to inhibit this with an IC_50_ > 600 μM (highest concentration tested) (data not shown). Also, LPS treatment significantly increased the levels of NF-κB levels in the nuclear fraction of LPS stimulated RAW 264.7 cells compared to untreated (normal) control. The molecules, NAT-1 and NAT-2 were found to be ineffective in reducing the levels of NF-κB in RAW264.7 cells stimulated with LPS when compared to LPS treated control group (**Figure 9**).

### Effect of NAT-1 and NAT-2 on Phosphorylation of AMPK, Akt, and p38 MAP Kinase in L6 Cells

**FIGURE 10:**
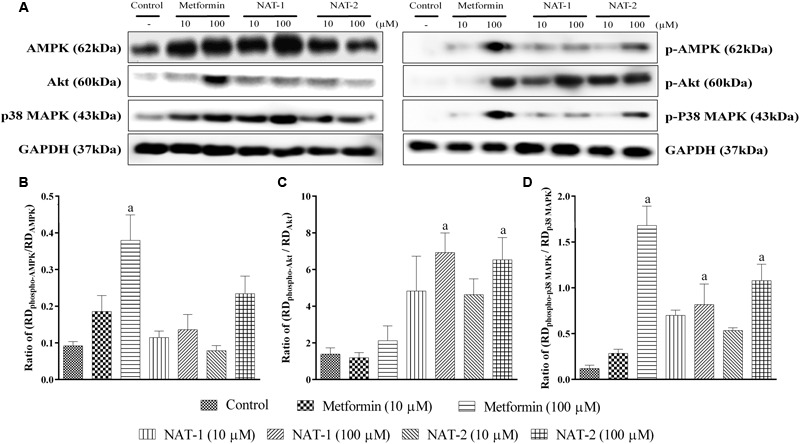
**Effect of NAT-1 and NAT-2 on phosphorylation of molecular markers – AMPK, p38 MAP kinase and Akt in L6 cells, where** **(A)** representative blots showing the levels of various markers and their phosphorylated forms tested; **(B)** ratio of relative densities of phospho-AMPK and AMPK; **(C)** ratio of relative densities of phospho-p38 MAPK and p38 MAPK; **(D)** ratio of relative densities of phospho-Akt and Akt. RD represents ratio of band intensity of marker protein to its respective housekeeping protein, i.e., GAPDH. Values are expressed as mean ± SEM. ^a^*p* < 0.05 vs. normal control.

Immunoblotting results from metformin (100 μM) pre-treated L6 myotubes revealed that the standard drug significantly increased phosphorylation of AMPK (at Th172) and p38 MAP kinase when compared to the respective untreated controls (**Figures 10A–C**).

Treatment with NAT-1 and NAT-2 (both 10 and 100 μM doses) did not increase the levels of phosphorylated AMPK, as compared to the control (**Figure 10B**).

However, NAT-1 and NAT-2 at 100 μM showed a significant increase in phosphorylated Akt and p38 MAP kinase levels (**Figures 10C,D**).

## Discussion

Previously, we had demonstrated the antidiabetic potential of NAT-1 and NAT-2 in STZ-induced diabetic mice (Kishore et al., 2009). However, the model of hyperglycaemia induced by injection of STZ has several limitations, viz. progression of mild hyperglycaemia into frank Type I diabetic condition, spontaneous recovery of animals, poor resemblance of the model with human Type II diabetes etc. STZ may be more suitable for developing a rodent model resembling human Type I diabetes, screening newer formulations of insulin, beta cell transplantation studies, and for treatments that may prevent beta cell death (King, 2012). In this regard, experiments were conducted to assess the antidiabetic activity of NAT-1 and NAT-2 in a diet induced pre-diabetic model in mice.

Metabolic effects of chronic high carbohydrate diet manifest as weight gain, glucose intolerance, insulin resistance, and hypertrophy and hyperplasia of β-cells (Ramirez et al., 1990). But frank hyperglycaemia is usually not observed. Our attempts in developing a model of diabetes by feeding HSD (30–60% sucrose) over a period of 3, 6, 9, 12, and 15 months in Swiss albino mice yielded a consistent pre-diabetic condition in those animals which were fed HSD (55% sucrose) for 15 months. In the present study, a high carbohydrate diet containing higher proportions of sucrose resulted in increased weight gain, glucose intolerance, impaired lipid profile and frank hyperglycaemia which may be the result of maintaining the animals for a longer period on HSD.

Treatment with NAT-1 and NAT-2 reduced the glucose intolerance as is evident from the AUC of the OGTT curve, the activity of NAT-1 being comparable to that of the standard drug metformin. The molecules also significantly reduced the elevated plasma triglyceride levels but elevated total cholesterol levels. However, a significant rise in the HDL cholesterol levels with NAT-1 was observed and we assume that the rise in total cholesterol levels could have been in part due to the increase in HDL levels. The rise in total cholesterol with NAT-2 is consistent with previous reports but a satisfactory explanation cannot be given at this stage. The structures of both NAT-1 and NAT-2 possess nicotinamide moiety which could have contributed to the rise in HDL cholesterol but further pharmacokinetic studies on the metabolism of the molecules are needed to confirm this. Despite treatment with NAT-1 and NAT-2 for a period of 45 days, the biochemical profile of the liver and kidney markers were largely unaffected. This was additionally confirmed as no major histological changes were present in liver and kidney tissues.

In the previous studies, the compounds NAT-1 and NAT-2 had shown increased glucose uptake and caused beta cell regeneration in an STZ model (Kishore et al., 2009). Further, NAT-1 exhibited a dose-response effect on hyperglycaemia (Karot et al., 2015). Therefore, to explore the underlying mechanism, *in vitro* experiments were designed in L6 myotubes on AMPK, Akt and p38 MAP kinase pathways.

In the studies on L6 myotubes, we have demonstrated a rise in phosphorylated AMPK levels with metformin which is in line with published reports. Also, the drug was found to increase the phosphorylation of p38 MAP kinase. Both the test molecules, NAT-1 and NAT-2 at 100 μM significantly increased the phosphorylation of Akt and MAP kinase (**Figure 10**). This indicates a possible activation of the PI3/Akt pathway which could have partly contributed to their anti-hyperglycaemic activity *in vivo*. It cannot be confirmed whether the phosphorylation of Akt by the test molecules is due to any direct action on Akt or by some indirect action that leads to phosphorylation of Akt. Further studies are required to confirm this.

Clinically used thiazolidine-2,4-diones, acting through PPAR-gamma receptor activation, play a vital role in pancreatic beta cell protection and preserves the islets from apoptosis (Kanda et al., 2010). The mechanisms involved in the protective effect by thiazolidine-2,4-diones also involve inhibition of cytokine production through NF-κB signaling (Remels et al., 2009). In the present study, the investigational thiazolidinon-4-one derivatives (NAT-1 and NAT-2) failed to inhibit the generation of ROS and nitrite, and increased the nuclear levels of NF-κB in LPS stimulated RAW 264.7 cells. Therefore, the previously reported pancreas protection by these molecules could not have been due to any anti-inflammatory properties. However, the test molecules activated p38 MAP kinase, a regulator of gene expression and cell survival. This could have contributed to the β cell protection (Kishore et al., 2009).

In summary, the anti-hyperglycaemic and hypolipidemic molecules, NAT-1 and NAT-2, could not activate AMPK. Both the molecules were able to phosphorylate Akt and p38 MAP kinase which could partially be responsible for their pharmacological actions.

Both the molecules have a thiazolidin-4-one moiety in their structure, a possible action on peroxisome proliferator activated receptor-gamma (PPAR-γ), a transcription factor activated by thiazolidinedione category of anti-diabetic drugs like pioglitazone, should be assessed by evaluating the target gene products of PPAR-γ. Also, the nicotinic acid moiety in the structure extends the possibility that the molecules could be interacting with members of nicotinic acid receptor family. Research on such lines could also be undertaken in future.

## Author Contributions

The study was conceptualized and designed by AK, NGK, and KN. NGK, AK, and AJ were involved in synthesis, purification and characterization of the test molecules. *In vivo* studies which also include feed preparation, animal maintenance and dosing were performed by JM, PN, and NtK. PS, NR, AS, KG, and GM were involved in *in vitro* studies that involved cell culturing, maintenance, estimations and western blot analysis. RS, CR were involved in data analysis and interpretation. All authors were involved in preparation of the manuscript and approved the final submission.

## Conflict of Interest Statement

The authors declare that the research was conducted in the absence of any commercial or financial relationships that could be construed as a potential conflict of interest.
